# Incidence and Mortality of Malignant Brain Tumors after 20 Years of Mobile Use

**DOI:** 10.3390/cancers15133492

**Published:** 2023-07-04

**Authors:** Mohy Uddin, Rozy Dhanta, Thejkiran Pitti, Diana Barsasella, Jeremiah Scholl, Wen-Shan Jian, Yu-Chuan (Jack) Li, Min-Huei Hsu, Shabbir Syed-Abdul

**Affiliations:** 1 Research Quality Management Section, King Abdullah International Medical Research Center, King Saud bin Abdulaziz University for Health Sciences, Ministry of National Guard—Health Affairs, Riyadh 11481, Saudi Arabia; drmohyuddin@yahoo.com; 2 Faculty of Management Sciences and Liberal Arts, Shoolini University of Biotechnology and Management Sciences, Solan 508976, India; roseydhanta@shooliniuniversity.com; 3 International Center for Health Information Technology (ICHIT), College of Medical Science and Technology, Taipei Medical University, Taipei 110, Taiwan; thejkiran.tn@gmail.com (T.P.); diana.barsasella5@gmail.com (D.B.); jj@tmu.edu.tw (W.-S.J.); 4 Graduate Institute of Biomedical Informatics, College of Medical Sciences and Technology, Taipei Medical University, 15F., No. 172-1, Sec. 2, Keelung Rd., Da’an Dist., Taipei 110, Taiwan; jaak88@gmail.com; 5 Department of Medical Records and Health Information, Health Polytechnic of Health Ministry Tasikmalaya, Tasikmalaya 6574, Indonesia; 6 AESOP Technology, San Francisco, CA 9876, USA; jeremiah@aesoptek.com; 7 School of Gerontology Health Management, College of Nursing, Taipei Medical University, Taipei 110, Taiwan; 8 Research Center for Artificial Intelligence in Medicine, Taipei Medical University, Taipei 110, Taiwan; 9 School of Health Care Administration, College of Management, Taipei Medical University, Taipei 110, Taiwan; 10 Department of Dermatology, Taipei Municipal Wan Fang Hospital, Taipei 110, Taiwan; 11 Graduate Institute of Data Science, College of Management, Taipei Medical University, 15F., No. 172-1, Sec. 2, Keelung Rd., Da’an Dist., Taipei 110, Taiwan; 12 Department of Neurosurgery, Wan-Fang Hospital, Taipei Medical University, Taipei 110, Taiwan

**Keywords:** malignant neoplasms, brain tumors, mobile phone use, epidemiologic studies, radiofrequency, electromagnetic field, joinpoint regression

## Abstract

**Simple Summary:**

This population-based study, spanning 20 years, revealed trends regarding the incidence and mortality due to malignant neoplasm of the brain (MNB) in association with mobile phone usage in Taiwan. The findings indicate a trend of increase in the number of mobile phone users over the study period, accompanied by a slight rise in the incidence and death rates of MNB. The compound annual growth rates further support these observations, highlighting consistent growth in mobile phone users and a corresponding increase in MNB incidences and deaths. While this study suggests a weak association between mobile phone users and MNB incidence and mortality, it is important to acknowledge that conclusive results cannot be drawn at this stage. Further investigation is required to obtain more definitive findings. Continued research in this area will contribute to better understanding of the potential risks and aid in the development of safer mobile phone usage practices in the future.

**Abstract:**

(1) Objective: This population-based study was performed to examine the trends of incidence and deaths due to malignant neoplasm of the brain (MNB) in association with mobile phone usage for a period of 20 years (January 2000–December 2019) in Taiwan. (2) Methods: Pearson correlation, regression analysis, and joinpoint regression analysis were used to examine the trends of incidence of MNB and deaths due to MNB in association with mobile phone usage. (3) Results: The findings indicate a trend of increase in the number of mobile phone users over the study period, accompanied by a slight rise in the incidence and death rates of MNB. The compound annual growth rates further support these observations, highlighting consistent growth in mobile phone users and a corresponding increase in MNB incidences and deaths. (4) Conclusions: The results suggest a weaker association between the growing number of mobile phone users and the rising rates of MNB, and no significant correlation was observed between MNB incidences and deaths and mobile phone usage. Ultimately, it is important to acknowledge that conclusive results cannot be drawn at this stage and further investigation is required by considering various other confounding factors and potential risks to obtain more definitive findings and a clearer picture.

## 1. Introduction

From the time of its inception in the mid-1980s, there has been a tremendous amount of increase in the use of mobile/smartphones across the globe, especially in recent decades. Though their unprecedented need and utility cannot be denied in our day-to-day lives, their advantages are threatened by the claims of their radiation effects on human health. Mobile phones are one of the most common electronic devices emitting Radio Frequency (RF) waves, which are used in close proximity to humans [1]. These RF radiations primarily target the brain as the main target organ of exposure and are linked with an increased risk for brain tumors [2,3]. The International Agency for Research on Cancer (IARC) classified RF electromagnetic non-ionizing radiations as ‘possibly carcinogenic to humans’ in 2011 [4]. 

Cancer is the second leading cause of deaths globally, as almost one in six deaths occur due to cancer, and the incidences of all types of cancers are rising on a global basis [5]. In Taiwan, cancer has been the leading (no. one) cause of the death for the last 41 years and is followed by heart diseases and then COVID-19 [6]. The overall incidence rate of cancer has gradually increased with the passage of time in the past years in Taiwan [7,8]. Primary malignant brain tumors are considered the leading cause for more than one-third of all brain tumors and are heavily associated with high morbidity as well as mortality [9,10,11]. Malignant neoplasm has become one of the leading causes of death in Taiwan since 1982 [12]. 

In the literature, a number of studies have been conducted to investigate the increasing trends of malignant brain tumors and their link with the use of mobile phones. For example, a UK-based study conducted over the period of 1995–2015 found the percentage rise for the Glioblastoma Multiforme Incidence across different age groups and suggested that widespread adverse environmental or lifestyle factors may be responsible [13]. Similarly, two more studies utilizing the Swedish National Inpatient Register (IPR) analyzed the rates of brain tumors with the use of phones in 1998–2013 and then in 2014–2015 and reported the increasing rates of tumors of unknown type but advised that the results should be used with caution due to the underreporting of brain tumor cases [2,14]. A multi-center case-control study (CERENAT) in France during 2004–2006 analyzed the association between mobile phone exposure and primary central nervous system tumors and found no significant increase in brain tumors with regular mobile phone users versus non-users; however, they reported a positive association for the heaviest users when considering the long-life cumulative duration and number of calls for gliomas [15]. A US study conducted using the data collected by the Surveillance, Epidemiology, and End Results (SEER) Program for brain cancer patients during 1977–2006 showed a slight rise in the incidence of brain cancer when there was a sharp rise in mobile phone use for a certain period of time, but overall, its concluding incidence data did not support the view of mobile phone use causing brain cancer [16]. Another population-based ecological study in Australia conducted during 1982–1992, 1993–2002 and 2003–2013 analyzed the trends of brain cancer using the data from the Australian Institute of Health and Welfare (AIHW) showed the possibility of a small risk or a latency period for more than 15 years, but its final results showed no increase in brain tumors due to the use of mobile phones [3]. Hardell et al. [17] performed a pooled analysis of two case-control studies on patients with diagnosed malignant brain tumors and one case-control study on deceased patients and controls diagnosed in the period covering 1997–2003, and the outcomes showed increased risk with a latency period and cumulative use in hours for mobile phones. The INTERPHONE study [18,19,20], conducted in 13 countries to analyze brain tumor risk linked with mobile phone usage, showed no increase in risk of glioma or meningioma with the use of mobile phones. The stable or declining incidence of brain tumors was recorded by some of the research studies in Nordic countries [21] and the US [22]. Research studies from the Nordic countries covering the period of 1979–2008 [21] and the US covering the period of 1997–2008 [22] were involved in analyzing latency scenarios relating mobile phone usage and their associated risk to develop brain cancers. The Nordic countries study did not observe a clear change in trends of glioma incidence rates. The US-based study reported that the risks of glioma with the use of mobile phones reported by one of the Swedish studies [17] and another Interphone study [18], which formed the basis of the IARC’s re-evaluation of mobile phone exposure, were not consistent with analyzed incidence trends in the US population data. The IARC rated this type of exposure as a possible human carcinogen (Grade 2B) [23]. The general public showed their concerns for the IARC non-conclusive report and with the media publishing contradictory information based on studies conducted by various research groups. 

Most recently, the International Commission on Non-Ionizing Radiation Protection (ICNIRP) published updated guidelines on RF radiation (100 kHz to 300 GHz) in terms of cancer risks in 2020 [24] and concluded that “In summary, no effects of RF EMFs on the induction or development of cancer have been substantiated”. But according to the experts from the Swedish and Estonian group of Hardell et al. [25] in 2021, the ICNIRP’s conclusion is incorrect and is in contradiction with the existing scientific evidence because there is an abundance of available convincing evidence of increased cancer risk and other negative health effects. 

Understanding the determinants of cancer is important for the development of strategies that can reduce the mortality and support the routine work of clinical staff involved in treating and educating patients. A recent review article evaluated evidence from population-based studies and concluded that most of them did not associate increased brain or salivary gland tumor risks with the use of mobile phones, but there were some uncertainties about long latency periods (>15 years), the subtypes of rare brain tumors, and usage of mobile phones in childhood [26]. Another Canadian study that used the data from the Canadian Cancer Registry and national industry statistics from 1992 to 2015 inferred that there was an absence of a rise in incidence rates of glioma with respect to the increased usage of cell phones, thus there might not be a causal link between cell phones and gliomas [27]. Likewise, one large-scale UK-based prospective study analyzed 1.3 million women’s data and came up with the accumulating evidence that the utilization of cell phones in normal circumstances does not increase brain tumor incidence [28,29]. Similarly, the results of another large-level Danish retrospective cohort study using 13 years of data from the Danish Cancer Registry, i.e., 1982–1995 and then followed-up to 21 years, i.e., 2002, did not support the hypothesis of an association between mobile phone use and the incidence of brain tumors, leukemia, and other cancers [30,31]. 

As a whole, the literature in this context is very wide and has mixed reviews about the connection of mobile phone usage with the development of brain tumors and death. According to the National Cancer Institute (NCI), there are a few studies that have shown some kind of evidence for the association of mobile phone use and brain tumor risk in humans, but most of the studies have suggested no association between them due to different discrepancies, such as ‘recall bias, inaccurate reporting, morbidity and mortality, participation bias, changing technology, exposure assessment limitations, insufficient follow up of highly exposed populations, inadequate statistical power/methods, and consideration for the chance of apparent effects’ [32].

Our study focused on the use of mobile phones only as the subject variable for investigating the incidence and mortality due to a malignant neoplasm of the brain (MNB), but in the literature, studies have identified various potential confounding factors and risk factors that could possibly be involved in the development of brain tumors and death. Cho et al. [33] described some confounding factors involved in malignant brain tumors that included: hereditary background, smoking status, alcohol intake, and obesity. Ostrom et al. [34] described that the brain tumors vary significantly with incidence by age, sex, race and ethnicity and listed few risk factors that are well validated, which include ionizing radiations, history of allergies, and genetic predisposition. In another review study, Vienne-Jumeau et al. [35] provided a review of various environmental risk factors for brain tumors and focused on ionizing radiations, toxic agents, exposure to chemicals, air pollution, and RF electromagnetic waves as the potentially involved risk factors. It also highlighted the important point that many environmental exposures have been investigated so far, but for most of them, the scientific evidence is still lacking.

Taiwan is one of the countries in the world which had an early adoption of mobile phones, with the highest percentage of users, where its subscription crossed more than 100% by the year 2002 [36,37,38]. A previous study was conducted in Taiwan to investigate the association between mobile phone usage and MNB incidences and deaths in 2000–2009 [38]. The results of this study did not detect any correlation between the morbidity/mortality of malignant brain tumors and mobile phone use in Taiwan. Because some cancers have a longer latency period, another study for a long period seemed to be useful. Hence, as a follow-up of previous study, we repeated a similar analysis for another decade, 2010–2019, in this current study.

## 2. Materials and Methods

A population-based study was performed to examine the association between mobile phone usage and MNB incidences and deaths for a period of 20 years (January 2000 to December 2019). The data for the incidence and deaths due to MNB were obtained from Taiwan Cancer Registry, and the mobile phone users’ data was collected from the National Communications Commission. 

Because the variables are count variables, linear regression was used to indicate whether mobile phone users were statistically significant as a predictor of incidence of MNB and deaths due to MNB. Liner regression was used for the interpretation of the relationship between the significance of the number of mobile phone users and the number of MNB incidences and deaths.

Apart from baseline regression, joinpoint regression was used to examine the trends of the number of mobile phone users on the number of incidences of MNB and the number of deaths due to MNB. Because the data were count data, annual average percentage change (AAPC) was calculated each year. An increasing change from the previous year was termed as 1 and a decreasing change from the previous year was termed as 0. The joinpoint regression was calculated and 95% confidence intervals were calculated by using Joinpoint Regression Software 4.9.1.0.

Regression: Regression was used to indicate whether mobile phone users were statistically significant as a predictor of the incidence of MNB and deaths due to MNB. It was based on the natural log. Equations are explained in the analysis.

$$Y_{i}=\int (X_{i}+\beta )+e_{i}$$

where *Y_i_* = dependent variable; *X_i_* = independent variable; *β* = unknown parameter; and *e_i_* = error terms.

The test for the overall significance of the regression was as follows.

An F-test was used to test the significance of the combined effect of the explanatory variables on dependent variables (Gujarati, 1988). The test is outlined as follows:

$$F=\frac{R^{2}\left(n-k\;\right)}{\left(1-R^{2}\right)\;\left(k-1\;\right)}$$

The statistical test of the significance of estimates was as follows.

Student’s *t*-test was applied to test the significance of estimates (Gujarati, 1988). The t-statistics were defined as follows:

$$t\;=\;\frac{b_{i}-b_{i}}{SE\;\left(b_{i}\right)}\;\mathrm{with}\;n–k\;\mathrm{degree}\;\mathrm{of}\;\mathrm{freedom}$$

where *b_i_* = least square estimates of *b_i_*; *b_i_* = hypothesis value of *b_i_* (Ho: *b_i_* = *b_i_*); *SE* (*b_i_*) = standard error of *b_i_*; *n* = sample size; and *k* = number of estimated parameters.

Joinpoint Regression: The joinpoint regression was used to find the best-fit line through several years of data; however, the joinpoint program used an algorithm that tests whether a multi-segmented line is a better fit than a straight or less-segmented line. Joinpoint explained the data trends by connecting various line segments on a log scale at joinpoints. Analysis began with the minimum number of joinpoints, i.e., 0, which represents a straight line and can fit up to a maximum of four joinpoints. Tests of significance used a Monte Carlo permutation method. In addition, an annual percent change (APC) in ASRs for each line segment and the corresponding 95% confidence interval were estimated. The APC was tested to determine whether a difference exists from the null hypothesis of no change (0%). In the final model, each joinpoint informed a slight change in the trends (increase or decrease) and each of those trends was described by an APC.

## 3. Results

**Figure 1:**
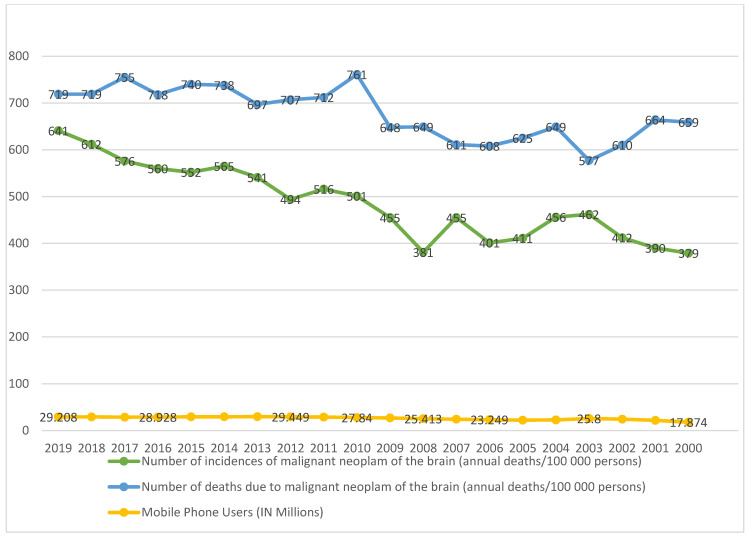
Diverging stack bar plot of MNB incidence/death rate vs. number of mobile phone users from 2000 to 2019 (source of data: National Communications Commission (2021) and Taiwan Cancer Registry (2021) in Taiwan).

The analysis of data showed the mean comparison of mobile phone users along with incidence of MNB and deaths due to MNB in Taiwan for the period of 20 years, i.e., January 2000 to December 2019, as shown in Table 1 and Figure 1. Figure 1 depicts a diverging stack bar plot of MNB incidence/death rate vs. number of mobile phone users from 2000 to 2019. As shown in Table 1, the average annual percentage change in the number of phone users (in millions) has increased by 63.41 (per 100,000 persons), while the number of incidences of MNB has shown a change of 9.10 (per 100,000 persons), and the number of deaths due to MNB has portrayed a change of 62 (per 100,000 persons) over the period of 20 years. Similarly, while calculating the compound annual growth rate, we found that there were growths of 1.95, 1.07, and 2.30 in the number of mobile phone users (in millions), the number of incidences of MNB (per 100,000 persons), and the number of deaths due to MNB (per 100,000 persons), respectively. This showed the increase in incidences and death rates which can be attributed to the positive growth rate of the number of mobile phone users.

### 3.1. Regression Analysis

The regression analysis was performed using two models, Model 1 and Model 2. Table 2 represents the linear regression results for our two models, i.e., Model 1 and Model 2. Model 1 considers INMB (number of incidences due to MNB (annual incidences/100,000 persons)) as the dependent variable and MOB (number of mobile phone users (in millions)) as the independent variable. The results show that the MOB coefficient slightly influences INMB and is at a significance level of 5%. With every increase of one million in the number of mobile phone users, there is an increase of 0.354 incidences of MNB per 100,000 users. The value of *R*^2^ (0.58) suggested that our independent variable (MOB) can explain 58% of the variations in the dependent variable, i.e., INMB.

Model 2 considers DMNB (number of deaths due to malignant neoplasm of the brain (annual deaths/100,000 persons)) as the dependent variable and MOB (number of mobile phone users (in millions)) as the independent variable. The results show that the coefficient of MOB is positive at the 1% significance level only. With every increase of one million in the number of mobile phone users, there is an increase of 0.957 per 100,000 users. The R2 value for Model 2 (0.631) suggested that our independent variable (MOB) can explain 63.1% of the variations in the dependent variable.

In Table 2, we present the results of our baseline regression model depicted in Equations (1) and (2)

(1) INMB=f(MNB)

(2) DMNB=f(MNB)

INMB = Number of incidences of malignant neoplasm of the brain (annual incidences/100,000 persons)

DMNB = Number of deaths due to malignant neoplasm of the brain (annual deaths/100,000 persons)

MOB = Number of mobile phone users (in millions)

Equations (1) and (2) can be further modified by taking the Log

(3) $$LNINMB_{i}=\alpha +\beta_{1}LNMOB_{1}+\varepsilon$$

(4) $$LNDMNB_{i}=\sigma +\beta_{2}LNMOB_{1}+\theta$$

LNINMB = Log of number of incidences of malignant neoplasm of the brain (annual deaths/10,000 persons)

LNDMNB = Log of number of deaths due to malignant neoplasm of the brain (annual deaths/10,0000 persons)

LNMOB = Log of number of mobile phone users (in millions)

**Figure 2:**
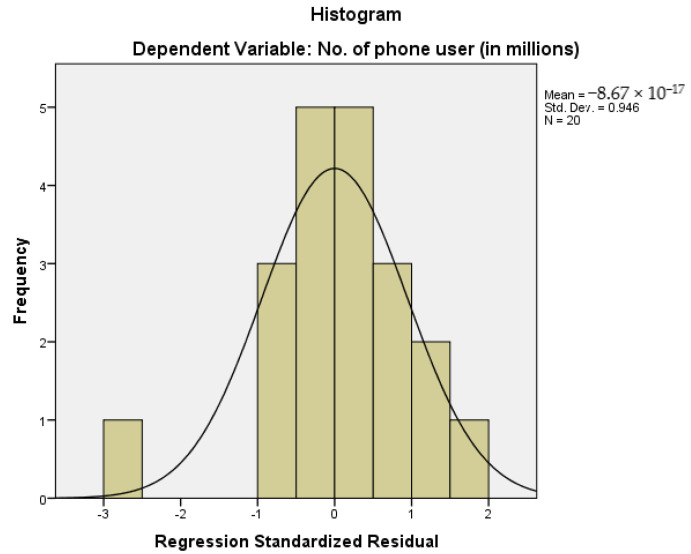
Histogram Representing the Observation of Dependent Variables.

For checking the distribution of variables, a histogram is presented in Figure 2 that shows observations of the dependent variable. As we can see from the histogram of Figure 2, the variables are normally distributed, and this means that our observations were balanced around the central tendency. The standard deviation value of 0.946 suggested that the observations were tightly packed around its central tendency, which means that the data observation does not have higher variability.

### 3.2. Joinpoint Regression Analysis

While fitting the joinpoint regression, two models were used:

First, Model A was used, with the log of the number of mobile phone users with the log of the number of incidences of malignant neoplasm of the brain (annual incidences/100,000 persons) during 2000 to 2019.

Second, Model B was used, with the log of the number of mobile phone users and the log of the number of deaths due to malignant neoplasm of the brain (annual deaths/100,000 persons) during 2000 to 2019.

**Figure 3:**
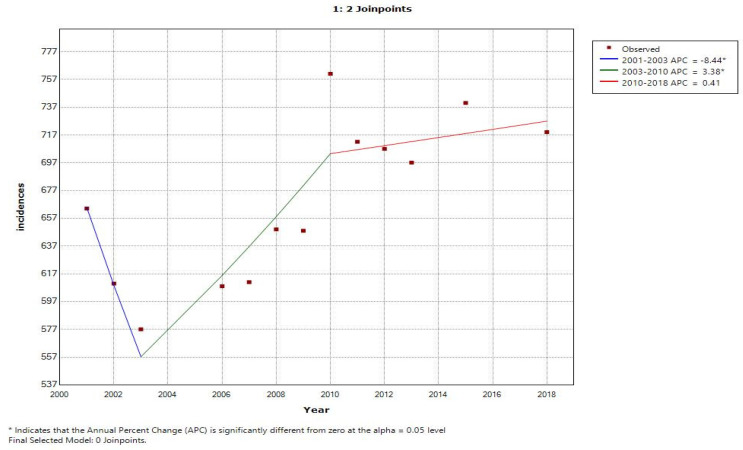
Joinpoint regression analysis of increasing number of mobile phone users with number of incidences of MNB (annual incidences/100,000 persons).

**Figure 4:**
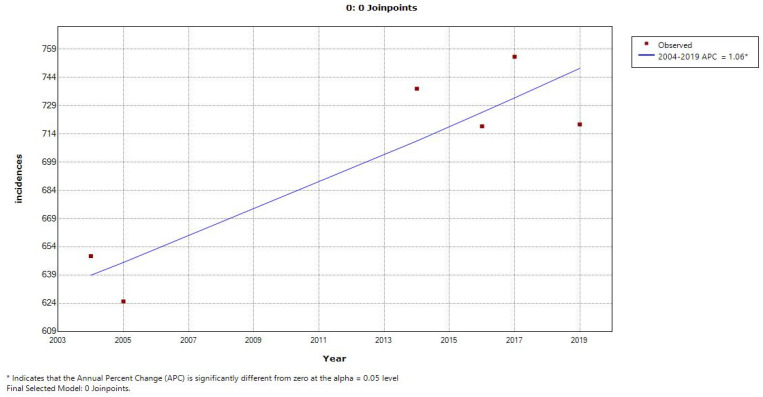
Joinpoints with decreasing number of mobile phone users and number of incidences of MNB (annual incidences/100,000 persons).

**Figure 5:**
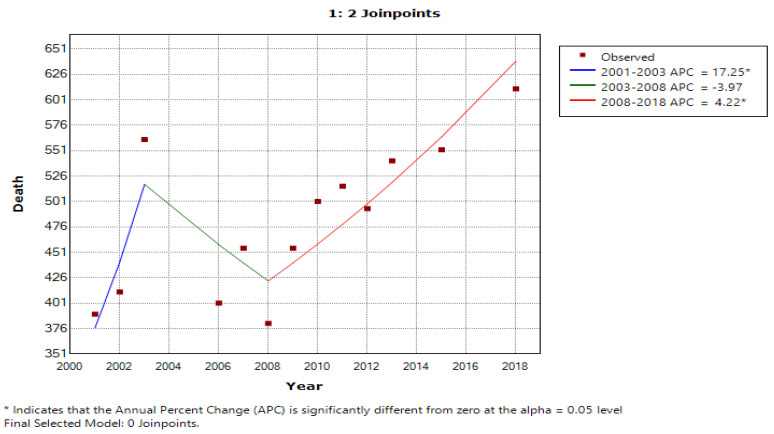
Joinpoint regression analysis of increasing number of mobile phone users with number of deaths due to MNB (annual deaths/100,000 persons).

**Figure 6:**
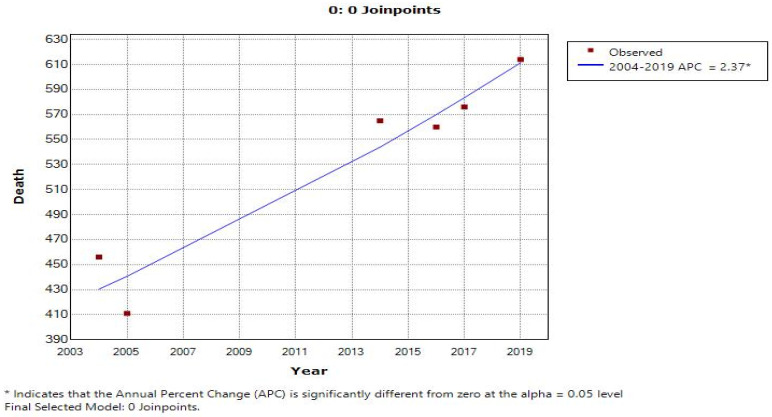
Joinpoints with decreasing number of mobile phone users with number of deaths due to MNB (annual deaths/100,000 persons).

In each model (Table 3 and Table 4), two charts are displayed which show the trends of incidence and deaths with an increase from the previous year in mobile phone users (depicted as 1) and a decrease in mobile phone users (depicted as 0) from the previous year. Further, Figure 3, Figure 4, Figure 5 and Figure 6 display the trends in incidence with increases and decrease s(from previous years) in the number of mobile phone users over 20 years, 2000–2019.

In Figure 3, the line graph displays two joinpoints (three line segments or trends), which shows that the trends for incidence rates slightly changed twice between 2000 and 2019. The annual percentage change was used to describe and test the statistical significance of the trends in the model. Table 3 shows that the incidence rate was not increasing and rather it was decreased by 8.4 percent until 2003; however, with the increase in mobile phone users, the incidence rate also increased by 3.4 percent during the years 2003 to 2018. Similarly, Table 4 depicts the trends of the number of mobile phone users with the number of deaths due to MNB (annual deaths/100,000 persons) during 2000 to 2019 for an annual increase in the percentage change of mobile phone users (1) and an annual decrease in the percentage change of mobile phone users (0). Figure 5, using a line graph, displays two joinpoints (three line segments or trends) and shows that the death rate trend slightly changed twice between 2000 and 2019. As per Table 4, we noticed that, with an increase in mobile phone users, the death rate until 2003 was increasing by 17.3 percent, while in the second period between 2003 and 2008, the death rate decreased by 4.0 percent and then again increased from 2008 to 2019 by 4.2 percent. With the decrease in number of mobile phone users, the incidence rate and death rate had a constant trend, with changes of 1.1 percent and 2.4 percent, respectively. The joinpoint analysis of the trends for the number of mobile users with incidence and death rates allows us to more accurately interpret the change over time and, more importantly, monitor the changes.

Table 4 shows the trends of the number of mobile phone users with the number of incidences of MNB (annual incidences/100,000 persons) during 2000 to 2019 for an annual increase in the percentage change of mobile phone users (1) and an annual decrease in the percentage change of mobile phone users (0) by joinpoint regression analysis, where APC stands for annual percentage change and AAPC stands for average annual percentage change. For detected joinpoints, years for locations were indicated and trends were given for two joinpoints with an increasing no. of mobile phone users, i.e., in 2003 at 3.4 and in 2010 at 0.4; with the decrease in mobile phone users, no joinpoints were detected.

Table 4 shows the trends in the number of mobile phone users with the number of deaths due to MNB (annual deaths/100,000 persons) during 2000 to 2019 for an annual increase in the percentage change of mobile phone users (1) and an annual decrease in the percentage change of mobile phone users (0) by joinpoint regression analysis, where APC stands for annual percentage change and AAPC stands for average annual percentage change. For detected joinpoints, years for locations are indicated and trends are given for two joinpoints with an increasing no. of mobile phone users, i.e., in 2003 at 4.8 and in 2008 at 4.2; with the decrease in mobile phone users, no joinpoints were detected.

Figure 3 shows the joinpoint regression analysis of an increasing number of mobile phone users with the number of incidences of MNB (annual incidences/100,000 persons). The line graph displays two joinpoints (three line segments or trends), which shows that the trends for incidence rates slightly changed twice between 2000 and 2019. The annual percentage change is used to describe and test the statistical significance of the trends in the model. Figure 5 shows the joinpoint regression analysis of an increasing number of mobile phone users with the number of deaths due to malignant neoplasm of the brain (annual deaths/100,000 persons). The line graph displays two joinpoints (three line segments or trends), which shows that the trends for the death rate slightly changed twice between 2000 and 2019. Figure 4 and Figure 6 show the calculation of the joinpoints for a decreasing number of mobile phone users with the number of incidences of MNB (annual incidences/100,000 persons) and the number of deaths due to MNB (annual deaths/100,000 persons), respectively. There are no such joinpoints seen in either of the cases, pertaining to fewer cases showing a decrease in mobile phone users (with previous years).

## 4. Discussion

The results of the regression analysis are depicted in two models: Model 1 (incidence), the number of mobile phone users with the number of incidences of MNB (annual incidences/100,000 persons) during 2000 to 2019; and Model 2 (deaths), the number of mobile phone users with the number of deaths due to MNB (annual deaths/100,000 persons) from 2000 to 2019. Model 1 depicted that the number of mobile phone users (in millions), the MOB coefficient, slightly influences the number of incidences of MNB (annual incidences/100,000 persons) and INMB at the 5% level. With every one-unit increase in mobile phone users, there is an increase of 0.354 incidences of MNB considering that the values are natural log values. Similarly, Model 2 considers the number of deaths due to MNB (annual deaths/100000 persons) and DMNB as the dependent variable, and the coefficient of the number of mobile phone users (in millions), MOB, is slightly changed at a 1% significance level. With every one-unit change in the percentage of the number of mobile phone users, we expect an increase of 0.957 percent in the number of deaths considering that the values are natural log values.

Moreover, the results of the joinpoint regression depicted that two joinpoints (three line segments) were found in Model 1; first in 2003 with an APC of −8.4 percent and 95% CI and second in 2010 with an APC of 3.4 (and then in 2018 with an APC of 0.4 and the highest AAPC was found in 2018), depicting the role of increasing mobile users in increasing incidences of MNB. Therefore, as we considered percentage change in our joinpoint regression, we can say that with every percentage change in the number of mobile phone users, there is an increase of 0.5 percent in incidences of MNB in 2018 (AAPC highest year). Similarly, in Model 2, again two joinpoints (three line segments) were seen, depicting that a joinpoint was found in 2003 with an APC of 17.3 and 95% CI, in 2008 with an APC of −4.0, and then in 2018 with an APC of 4.2 and 95% CI. The highest AAPC was found in 2019 for deaths. We can say that with every one-unit change in the percentage of the number of mobile phone users, we expect an increase of 2.4 percent in the number of deaths due to MNB.

We hypothesized two reasons behind the increased number of incidences and deaths due to MNB during the study period. First, the advancements in medical diagnostic technology, such as SPECT, PET, CT, MRI, and optimal imaging, etc., as shown in the literature [39,40,41,42,43], have increased the rates of diagnosis, as these technologies can now discern brain tumors with better accuracy, which could have otherwise remained undiagnosed in the past. Second, the increase in the aging population plays a role, as some of the recent research in the literature is based on animal experimental evidence and epidemiological studies [44,45,46], which support categorizing RF radiations as carcinogenic to humans. With the advent of 5G in telecommunication, there is a focus on increasing the number of base stations and making the frequency range higher than 3.5 GHz in order to provide better connectivity to mobile phones and Internet of Things (IoT) devices. On one side, the advancement of technologies has led to a drastic increase in the use of mobile phones, but on the other side, the technological evolution has also caused a decrease in RF EMFs emitted by these mobile phones [15]. It seems prudent to continue our work in the forthcoming decades among different age groups of the Taiwanese population. The claims of Morgan et al. indicated that the time duration required for the development of the diseases from the reported latency of known causes of brain cancer could range anywhere from 10 to 50 years [47].

Along with the multi-disciplinary studies on the effects of mobile phone radiation on humans, extensive studies on the usage of new generation technology are also taking place and gaining importance for their overall contribution to public wellbeing. The future research should focus on the very heavy cell phone users by considering the latest features of the continuously evolving wireless technology [28,29]. Moreover, it is important for the public to have awareness about the impact of mobile phones on human health; and policy makers and authorities also need to implement stringent radiation norms for public safety [1]. The use of wired or wireless headsets can reduce the RF exposure to the brain as they are not placed against the head, and the exposure also declines when mobile phones are used hands-free [48,49].

## 5. Limitations

Our study had some limitations, such as the following: the data that were used were from one region, the detailed characteristics of the mobile phone users were unavailable, and the study was limited only to the period of two decades. Apart from that, our study was limited to the use of mobile phones only as the subject variable for investigating the incidence of MNB and mortality due to MNB, but there might be many other confounding variables and risk factors that could possibly be involved in the development of brain tumors and death, primarily, for example, prolonged contact with ionizing radiation agents, not non-ionizing radiation, genetic predisposition, exposure to chemicals, and congenital diseases. In addition, adverse environmental factors and various lifestyle factors could also contribute in this context. Therefore, all of these factors must be considered for the exact and in-depth analysis of the root cause of the incidence of MNB and mortality due to MNB. In addition, as a limitation, our study did not explore the age-wise breakdown aspects of mobile users to ascertain their association with MNB, as the cancer cases usually have steep rise in those middle-aged and beyond like the elderly. Moreover, another important factor that needs to be looked at is the radiation exposure duration, which could provide a better understanding of the latency period of brain cancer in further studies. Meanwhile, potential precautionary measures should also be propagated to maintain the health of the Taiwanese population.

## 6. Conclusions

This population-based study, spanning 20 years, revealed trends regarding the incidence of MNB and mortality due to MNB in association with mobile phone usage in Taiwan. The findings indicate trends of increase in the number of mobile phone users over the study period, accompanied by a slight rise in the incidence and death rates of MNB. The compound annual growth rates further support these observations, highlighting the consistent growth in mobile phone users and a corresponding increase in MNB incidences and deaths. These results suggest a weaker association between the growing number of mobile phone users and the rising rates of MNB, and no significant correlation was observed between MNB incidences and deaths and mobile phone usage. In general, the literature in this context is very wide and has mixed reviews, but overall, the literature provides only a little evidence or does not show evidence of the association between mobile phone users and the development of brain tumors and death. Ultimately, it is important to acknowledge that conclusive results cannot be drawn at this stage; therefore, further investigation is required to obtain findings that are more definitive.

Considering the potential risks associated with emerging technologies such as 5G, which has higher data transmission rates and frequencies, it is crucial to examine radiation exposure levels and durations when assessing the association between mobile phone usage and cancer. Future research should explore different age groups of mobile phone users and investigate the duration of radiation exposure to gain a better understanding of their potential associations with MNB, as the latency period for brain cancer can range from 10 to 50 years. It is reassuring to know that engineers are actively working on reducing harmful radiation with the advancements of communication technologies. Continued research in this area will contribute to the better understanding of the potential risks and aids in the development of safer mobile phone usage practices in the future. Finally, further in-depth investigations for the other related confounding variables and risk factors that could be involved in the development of MNB are sine qua non for understanding the bigger picture.

## Figures and Tables

**Table 1:** Average annual percentage change (AAPC) and compound annual growth rate (CAGR) for the period of 20 years (2000–2019).

Item	No. of Mobile Phone Users	No. of Incidences of MNB	No. of Deaths Due to MNB
AAPC	63.41	9.10	62.00
CAGR	1.95	1.07	2.30

MNB: Malignant neoplasm of the brain.

**Table 2:** Numerical Measures and Statistical Significance between Mobile Phone Users and MNB Incidences and Deaths.

Factors	Model 1 (Incidence)	Model 2 (Deaths)
Constant	2.910 *	−3.542 *
MOB	0.354 *	0.9572 *
Std. Error.	0.115	0.172
*R* ^2^	0.58	0.631
Adjusted *R*^2^	0.343	0.610

*: *p*-valve 0.05; bold words to show they are only significant values.

**Table 3:** Depiction of joinpoint regression for number of mobile phone users with number of incidences of MNB (annual incidences/100,000 persons).

**Cohort**	**Annual Percent Change (APC)**	
	Upper Joinpoint	APC
**Increase (1)**	2003	−8.4 *
	2010	3.4
	2018	0.4
	Average Annual Percent Change (AAPC)	
	Upper Joinpoint	AAPC
	2018	0.5
* Indicates that the annual percent change (APC) is slightly different from zero at the alpha = 0.05 level		
**Decrease (0)**	Upper Joinpoint	APC
	2019	1.1 *
	Upper Joinpoint	AAPC
	2019	1.1 *
* Indicates that the average annual percent change (AAPC) is slightly different from zero at the alpha = 0.05 level		

**Table 4:** Depiction of joinpoint regression for number of mobile phone users with the number of deaths due to MNB (annual deaths/100,000 persons).

**Cohort**	**Annual Percent Change (APC)**	
	Upper Joinpoint	APC
**Increase (1)**	2003	17.3 *
	2008	−4.0
	2018	4.2 *
	Average Annual Percent Change (AAPC)	
	Upper Joinpoint	AAPC
	2019	3.2 *
* Indicates that the annual percent change (APC) is slightly different from zero at the alpha = 0.05 level		
**Decrease (0)**	Upper Joinpoint	APC
	2019	2.4 *
	Upper Joinpoint	AAPC
	2019	2.4 *
* Indicates that the average annual percent change (AAPC) is slightly different from zero at the alpha = 0.05 level		

## Data Availability

The raw data supporting the conclusions of this article will be made available by the authors upon request, without undue reservation.
